# Predicting cash holdings using supervised machine learning algorithms

**DOI:** 10.1186/s40854-022-00351-8

**Published:** 2022-05-18

**Authors:** Şirin Özlem, Omer Faruk Tan

**Affiliations:** 1 Department of Industrial Engineering, Faculty of Engineering, MEF University, Istanbul, Turkey; 2 Department of Accounting and Finance, Faculty of Business Administration, Marmara University, Istanbul, Turkey

**Keywords:** XGBoost, MLNN, Cash holdings, Turkey, Machine learning, C38, C53, G30

## Abstract

This study predicts the cash holdings policy of Turkish firms, given the 20 selected features with machine learning algorithm methods. 211 listed firms in the Borsa Istanbul are analyzed over the period between 2006 and 2019. Multiple linear regression (MLR), k-nearest neighbors (KNN), support vector regression (SVR), decision trees (DT), extreme gradient boosting algorithm (XGBoost) and multi-layer neural networks (MLNN) are used for prediction. Results reveal that MLR, KNN, and SVR provide high root mean square error (RMSE) and low R^2^ values. Meanwhile, more complex algorithms, such as DT and especially XGBoost, derive higher accuracy with a 0.73 R^2^ value. Therefore, using advanced machine learning algorithms, we may predict cash holdings considerably.

## Introduction

What are the motivations for firms to hold cash and cash equivalents? In other words, why do firms not use their cash to redistribute or reinvest? These questions are two of the most debated topics in the corporate finance literature. Firms have significantly increased their cash holdings over the past two decades, especially because it allows them to manage unforeseen cash flow changes, daily funding operations, and financing of long-term projects (Opler et al. 1999). However, firms must keep an appropriate amount of cash; holding too much causes managers to pursue their interests, resulting in shareholder losses and perhaps a financial crisis. The rate of return on corporate cash holding is typically lower than the market interest rate, raising the opportunity cost of cash holdings (Wu et al. 2021). According to two different approaches, holding an optimal cash amount is an essential subject in finance.

In the finance literature, four classes of motives are identified for firms to hold cash (Bates et al. 2009): *transaction*, *precaution*, *agency cost*, and *tax motive*. First, firms with insufficient internal finance can convert non-financial assets into cash, issue new shares and debt, or curtail dividend payments. However, firms want to avoid transaction costs, which produce the transaction motive. Miller and Orr (1966) documented that intermediation costs could tempt a firm to hold more liquid assets. The precautionary motive refers to cash reserves being kept as a precautionary motive against an unexpected shortfall or to capture lucrative investment opportunities (Bates et al. 2009; Keynes 1936; Kim et al. 1998). Firms that do not set aside funds for this purpose may be compelled to forego valuable investment projects or struggle against bankruptcy (Campello et al. 2010). The conflict of interest between managers and shareholders results in agency motives for keeping cash; managers prefer to use firm resources to meet their own interests rather than maximize shareholders’ benefits (Jensen and Meckling 1976). Entrenched managers tend to retain cash instead of making dividend payments to shareholders when facing negative investment projects. In this way, they increase assets under their control and have power over the firm’s investment decisions (Jensen 1986). When firms face greater repatriation taxes, they choose to keep ample cash abroad as a tax motive (Foley et al. 2007).

To determine the cash holdings behavior of firms, studies have used different financial variables at the firm level in the literature. Some variables used are as follows: size (Bigelli and Sánchez-Vidal 2012; Boubakri et al. 2013; Drobetz and Grüninger 2007; García-Teruel and Martínez-Solano 2008; Lozano and Yaman 2020; Ozkan and Ozkan 2004), leverage (Bigelli and Sánchez-Vidal 2012; Drobetz and Grüninger 2007; Ferreira and Vilela 2004; García-Teruel and Martínez-Solano 2008; Ozkan and Ozkan 2004; Uyar and Kuzey 2014), dividend (Bigelli and Sánchez-Vidal 2012; Song and Lee 2012), sales growth (Bigelli and Sánchez-Vidal 2012; Boubakri et al. 2013; Song and Lee 2012), net working capital (Bigelli and Sánchez-Vidal 2012; Boubakri et al. 2013; Diaw 2021; Lozano and Yaman 2020), cash flow (Boubakri et al. 2013; Diaw 2021; Ferreira and Vilela 2004; Lozano and Yaman 2020; Uyar and Kuzey 2014), capital expenditure (Boubakri et al. 2013; Diaw 2021; Uyar and Kuzey 2014), and tangibility (Drobetz and Grüninger 2007; Uyar and Kuzey 2014). With classical regression methods, the impact of many financial variables on the firms’ cash holdings behavior has been examined. Unlike the previous literature, we try predicting the cash holdings behavior of firms by applying advanced machine learning approaches to address the gap in the literature. Machine learning, which is one of the most popular data analysis methods nowadays, consists of algorithms that predict the outcomes as accurately as possible. Algorithms vary according to the type of data to be predicted. If the dataset contains a set of features that influence the outcome data and if the labeled outcome data are given, supervised learning algorithms are used. Moreover, the supervised learning algorithms are categorized based on the outcome data label (regression or classification). Cash forecasting is significant for determining the optimal cash holdings level. Machine learning can help firms predict or estimate their cash holdings level in the future. The cash forecast will assist managers in determining how the cash can be used to generate greater profit and how managers can protect the company from financial challenges (Donepudi et al. 2020). Moreover, machine learning techniques can be used for prediction and analysis instead of merely reporting numbers and statistics (Rafi et al. 2020).

The present study aims to predict the cash holdings policy of Turkish firms by applying various supervised machine learning regression methods individually starting from simple ones, such as linear regression, support vector regression (SVR), and k-nearest neighbor (KNN), and proceeding with more complex algorithms, such as extreme gradient boosting algorithm (XGBoost) and neural networks, respectively. 211 listed firms in the Borsa Istanbul are included in the study, and the time spans between 2006 and 2019. This study’s major contribution is filling the following gaps in the literature. First, most previous studies have employed regression analysis to predict cash holdings, and only a few studies use machine learning techniques. Second, to the best of the authors’ knowledge, this study is the first to predict cash holdings with machine learning algorithms in Turkey.

Our model has 19 financial ratios and Turkey’s country-specific World Uncertainty Index (WUI). The methods are evaluated based on RMSE and R^2^ metrics. Our main findings are as follows. The results show that less complicated multiple linear regression (MLR) and k-NN and SVR provide high RMSE and low R^2^ values. In contrast, more complex ones, such as decision trees (DT) and especially XGBoost, derive higher accuracy (e.g., 0.73 R^2^ value), which is a satisfactorily high value in finance. Pretax margin, net margin, cash flow, and current ratio are the most fundamental features providing a high-performance prediction model.

The remainder of this paper is organized as follows. Section 2 summarizes the literature review, and Sect. 3 explains the data and research methodology. Section 4 indicates the empirical results, and finally, Sect. 5 presents the conclusion and discussion part.

## Literature Review

In recent years, machine learning algorithms have been used in the corporate finance area. For instance, Wu et al. (2021) used the DT methods to predict the cash holdings of the high-tech industry in Taiwan by applying J48, logistic model tree (LMT), random forest (RF), REP tree, simple CHART, extra tree, and BF tree. Their findings revealed that RF has the best prediction rate of all the DT. Moubariki et al. (2019) analyzed the cash management of the public sector by applying DT, RF, and neural network. The study documented that the DT is the best prediction method. Meanwhile, Bae (2010) examined the forecasting dividend policy decisions of Korean firms using support vector machines (SVM), DT, and neural networks. Their results documented that SVM outperforms other techniques to forecast dividend policy. Abdou et al. (2012) predicted the share price and dividend yield performance of transportation globally from 2005 to 2012. They revealed that the generalized regression neural network performs well in minimizing errors and is better than the conventional regressions. Moreover, Won et al. (2012) analyzed dividend policy forecasting through genetic algorithm-based knowledge refinement (GAKK) and other models, such as CHAID, CART, QUEST, and C5.0. They found that GAKK is the best model to forecast the dividend policy. Gholamzadeh et al. (2021) predicted financial constraints for listed firms in Tehran Stock Exchange by applying the Gaussian process and radial neural network. They confirmed that machine learning methods are suitable for predicting financial constraints. The percentage of institutional ownership, return on assets, financial leverage, operating cash flow to assets, and the company's value are the main variables in predicting the financial constraints. Furthermore, Huang and Yen (2019) predicted financial distress for Taiwanese firms by applying supervised, unsupervised, and hybrid learning algorithms. Traditional SVM, hybrid associative memory with translation, hybrid genetic algorithm-fuzzy clustering, XGBoost, deep belief network (DBN), and the hybrid DBN–SVM models are included. Li et al. (2021) mentioned the challenges in determining the number of clusters for financial data due to its size and different distributions and interpreting the results. They proposed two models: the first model introduces a new cluster quality evaluation criterion and uses it for hyperellipsoidal cluster detection. The second one, a revised support vector data description model, is an optimization algorithm that makes clusters tighter and easily interpretable. The model is evaluated on various financial datasets and results in easier to interpret clusters. They documented that XGBoost provides more accurate financial distress prediction. Meanwhile, Wang (2017) predicted bankruptcy using SVM, neural network with dropout, and autoencoder. Among these, neural network with dropout has the highest accuracy. Also, these three models perform better than the former methods of logistic regression, genetic algorithm, and inductive learning. Many studies on bankruptcy prediction model benefit from accounting-based ratios. Unlike previous studies, Kou et al. (2021a, b) predicted bankruptcy for small and medium-sized enterprises (SMEs) in China that use transactional and payment network-based variables without the need for firms' financial data, including more than 240 million daily transactions. They found that payment and transactional data-based variables improve SMEs bankruptcy prediction and the ensemble model of XGB outperforms individual classifiers. Meanwhile, Mousa et al. (2021) used three supervised machine learning methods, namely, RF, quadratic discriminant analysis, and linear discriminant analysis, to predict the financial performance of 63 listed banks in emerging markets. They revealed that the RF method provides the best predictive models and that incorporating disclosure tone variables into the predictive model with financial variables enhances the accuracy and quality of these models. Ozgur et al. (2021) used machine learning techniques (i.e., XGBoost, regression tree, boosting, bootstrap aggregating, RF, and extra-trees) to predict bank lending behavior. They documented that RF is the best predictive model. Moreover, Abellán and Castellano (2017), Bequé and Lessmann (2017), and Harris (2015) predicted credit scoring by applying different machine learning methods. Popescu and Dragotă (2018), Wang (2017), and Zheng and Yanhui (2007) examined financial distress and bankruptcy by applying different machine learning algorithm models. Meanwhile, Kou et al. (2014) proposed approach that uses multiple criteria decision-making methods, k-means, COBWEB, expectation–maximization, repeated-bisection approach, graph-partitioning algorithm, and density-based methods to assess the quality of clustering algorithms in the domain of financial risk analysis. They examined German and Australian credit card application and Korean bankruptcy datasets. Their findings reveal that repeated-bisection approach outperforms other selected clustering algorithms. Basak et al. (2019) and Fiévet and Sornette (2018) forecasted stock prices based on XGBoost and found more accurate results. Furthermore, Bhambri (2011), Chen and Huang 2011, Chitra and Subashini (2013), and Hassani et al. (2018) employed machine learning algorithms for analyzing the banking sector. Kou et al. (2021a, b) evaluated fintech-based investments of European banking services by applying the IT2 fuzzy DEMATEL model to weight the criteria and the IT2 fuzzy TOPSIS method to rank the investment alternatives. They identified three financial criteria (i.e., cost management, sales volume, and increase in market value) and three non-financial criteria (i.e., customer satisfaction, competitive advantage, and organizational efficiency). Their results demonstrate that the competitive advantage is the essential factor among the fintech-based determinants. In contrast, sales volume has the weakest performance. Generally, non-financial factors are more significant than financial factors.

## Data and methodology

### Data

This study considers 211 listed firms in the Borsa Istanbul (BIST) from 2006 to 2019. Yearly firm-level data variables are obtained from Thomson Reuters DataStream. Turkey’s WUI data are taken from its website, and age data of firms are obtained manually from Google Search. The original sample was subjected to several sample selection parameters. The fiscal year of sports teams is different, and thus, they are excluded. Moreover, real estate investment trust firms are also excluded from the data because they have different financial variables items. Finally, firms in the financial sector, such as banks, insurance, leasing, factoring, and other firms related to financial institutions, are excluded because their accounting ratios are not comparable with the accounting ratios of other firms. Firms with missing data or negative leverage and tangibility in the sample are also excluded. Meanwhile, firms are included if they have at least four years of consecutive data. After data processing, we obtained 211 firms representing 2,408 firm-year observations. Table 1 displays the definition of each variable.

**Table 1:** Definition of variables and determinants factors of cash holdings

Explanatory variables	Definitions	Studies	Source
CASH	The ratio of cash and cash equivalents to the total assets		Thomson Reuters
DIV	The ratio of total dividend payments to the total assets	Bigelli and Sánchez-Vidal (2012), Bhuiyan and Hooks (2019), Song and Lee (2012), Wu et al. (2021)	As Above
SG	Annual change in sales growth (%)	Bigelli and Sánchez-Vidal (2012), Boubakri et al. (2013), Kim et al. (2021), Song and Lee (2012)	As Above
SIZE	Natural logarithm of total assets in current USD	Bigelli and Sánchez-Vidal (2012), Boubakri et al. (2013), Drobetz and Grüninger (2007), García-Teruel and Martínez-Solano (2008), Lozano and Yaman (2020), Ozkan and Ozkan (2004)	As Above
CAPEX	The ratio of capital expenditure to the total assets	Boubakri et al. (2013), Diaw (2021), Guney et al. (2007), Uyar and Kuzey (2014)	As Above
CF	The ratio of the sum of pre-tax income plus depreciation to the total assets	Boubakri et al. (2013), Diaw (2021), Ferreira and Vilela (2004) Guney et al. (2007), Lozano and Yaman (2020), Uyar and Kuzey 2014), Wu et al. (2021)	As Above
IE	The ratio of interest expense to the total assets	Schauten et al. (2011)	As Above
NWC	The ratio of non-cash working capital to the total assets	Bigelli and Sánchez-Vidal (2012), Boubakri et al. (2013), Diaw (2021), Lozano and Yaman(2020)	As Above
TANG	The ratio of net fixed assets to the total assets	Bhuiyan and Hooks (2019), Drobetz and Grüninger (2007), Uyar and Kuzey (2014)	As Above
STD	The ratio of short-term debt to the total assets	Benkraiem et al. (2020), Lozano and Yaman(2020)	As Above
ROA	The ratio of net income to the total assets	Batuman et al. (2021), Bhuiyan and Hooks (2019), Cai et al. (2016), Cambrea et al. (2021), Sarfriz et al. (2020)	As Above
ROE	The ratio of net income to the total equity	Manoel et al. (2018)	As Above
AR	The ratio of account receivable to the total assets	Mohammadi et al. (2018), Wu et al. (2012)	As Above
AP	The ratio of accounts payable to the total assets	Chen et al. (2014), Wu et al. (2012)	As Above
CR	The ratio of current assets to current liabilities	Manoel et al. (2018), Ozkan (2001)	As Above
EPS	Earnings per share	Sarfriz et al. (2020)	As Above
ROIC	The ratio of net operating profit after tax to the total assets	Sarfriz et al. (2020)	As Above
NET MARGIN	The ratio of net income to the net sales	Angelovska and Valentinčič (2019)	As Above
PRETAX MARGIN	The ratio of profit before tax to the net sales	Mihai et al. (2018)	As Above
AGE	The foundation year of the firm	Bigelli and Sánchez-Vidal (2012), Cai et al. (2016), Gao et al. (2013), Manoel et al. (2018), Wu et al. (2021)	Google Search
WUI_TURKEY	Annual average of quarterly data of World Uncertainty Index		https://worlduncertaintyindex.com/

### Methodology

Recently, machine learning algorithms have frequently been used as prediction tools even in finance, especially for price prediction, financial risk management, financial services, and decision making (Xiao and Ke 2021). To predict bank lending, we used and compared various machine learning algorithms, such as panel regression, tree regression, RF, and XGBoost (Ozgur et al. 2021). Moreover, on-site supervision and self-supervision approaches are compared using machine learning approaches like the RF algorithm (Antunes 2021). In the cryptocurrency field, machine learning-based approaches, such as SVM and RF, are used for trading strategies (Sebastiao and Godinho 2021). RF and long short-term memory, which is a deep learning method, are combined to analyze the effect of COVID-19 on bank regulations (Polyzos et al. 2021). We explained various machine learning regression methods used in this study in the following.

#### Multiple linear regression

This method is the extended version of simple linear regression with the formula shown in [1].

1 $${\varvec{Y}} = {\varvec{\beta}}_{0} + {\varvec{\beta}}_{1} {\varvec{X}}_{1} + {\varvec{\beta}}_{2} {\varvec{X}}_{2} + \cdots + {\varvec{\beta}}_{{\varvec{k}}} {\varvec{X}}_{{\varvec{k}}} + \user2{\varepsilon }$$

This formula is the vectorized form for n data values, where Y: response (target) variable as a vector of n values, X_k_: *k*th explanatory variables (each k element as a vector of n values), ${\upbeta }_{0}$: constant (the value for y-intercept), ${\upbeta }_{{\text{k}}} :$ slope coefficient for kth explanatory variable, ${\upvarepsilon }$: Error term of the model.

The following five assumptions should be satisfied to apply a multiple regression model:

1. A linear relationship exists between each explanatory variable and the response variable. This relationship might be checked using scatter plots.
2. The dataset has no or negligible multicollinearity issue. Multicollinearity represents the collinearity among explanatory variables (features). To check multicollinearity, scholars have commonly used variance inflation factor (VIF).
3. Model residuals were normally distributed. Q-Q plots are frequently used to check the normality.
4. The data values in the dataset have no or negligible autocorrelation.
5. The residual variances are constant (homoscedasticity).

After checking these assumptions, we ran and evaluated the model based on some performance measures, such as mean square error, RMSE, and/or the coefficient of determination (R^2^).

#### K-nearest neighbors regression

The KNN algorithm is mostly used for classification, yet it can also tackle regression problems. The KNN regression algorithm starts by defining the distances between each observed data value (with the given features) and the new data value with the unknown target. The distance metrics are either Euclidean or Manhattan distance functions (Zhang 2016). In n-dimensional space, the Euclidean distance between two points $p\left( {p_{1} , \ldots ,p_{n} } \right)$ and $q\left( {q_{1} , \ldots ,q_{n} } \right)$ is calculated using [2]:

2 $${\varvec{d}}\left( {{\varvec{p}},{\varvec{q}}} \right) = \sqrt {\left( {{\varvec{p}}_{1} - {\varvec{q}}_{1} } \right)^{2} + \cdots + \left( {{\varvec{p}}_{{\varvec{n}}} - {\varvec{q}}_{{\varvec{n}}} } \right)^{2} }$$

Moreover, the Manhattan distance function is about the absolute differences of the points [3]:

3 $${\varvec{d}}\left( {{\varvec{p}},{\varvec{q}}} \right) = \mathop \sum \limits_{{{\varvec{i}} = 1}}^{{\varvec{n}}} \left| {{\varvec{p}}_{{\varvec{i}}} - {\varvec{q}}_{{\varvec{i}}} } \right|$$

Next, parameter k, which is the number of neighbor points, is considered before assigning any new data value. Low values of parameter k might cause overfitting, whereas high values might cause high model errors both in the training and test data. After that, the average of k closest data values is assigned as the unknown target value. Grid search cross-validation, which is a technique to determine the optimal hyperparameters in the selected model, is often applied to find the best k value. The next step is to find the loss function between the assigned dependent value and the corresponding actual dependent variable value (i.e., CASH values for different observations). The overall loss function is minimized in the training phase, and the result is reflected in the model settings.

### Support vector regressor

This method is another simple-to-apply algorithm designed by Vapnik (1995). Unlike the multiple regression method, which tries minimizing the error between the actual target value and the predicted target value, SVR finds the best decision boundary, called hyperplane, within a threshold value. This is the distance of each target value to an epsilon value, or the maximum error:

4 $$\left| {{\varvec{y}}_{{\varvec{i}}} - {\varvec{wx}}_{{\varvec{i}}} } \right| \le \user2{\varepsilon }$$

In this formula, y is the actual dependent value, and ${\varvec{wx}}_{{\varvec{i}}}$ is the fitted model value. Therefore, the method is flexible (flexibility in setting a threshold value) compared with linear regression. One critical hyperparameter in this method is the regularization (i.e., the technique to minimize overfitting) parameter C. Grid search cross-validation is often applied to find the best C value.

### Decision trees

A DT is a tree-structured method used for classification and regression problems. This method cuts down a dataset into smaller parts while developing an associated DT. Determining the terms “entropy” and “information gain” for DT applications is critical.

Entropy H is a metric for the uncertainty of a probability distribution p, as displayed in [5]:

5 $$H\left( p \right) = H\left( {p_{1} , \ldots ,p_{n} } \right) = - \mathop \sum \limits_{i = 1}^{n} p_{i} *\log_{2} p_{i}$$

which is tried to be minimized (Ertel 2017). Meanwhile, information gain (IG) is the metric that depicts the reduction (improvement) in entropy in X after splitting the dataset regarding feature (variable) Y. It is calculated as follows:

6 $$IG\left( {X; Y} \right) = H\left( X \right){-}H(X | Y)$$

The dataset is partitioned with respect to the highest IG; therefore, DT algorithms work top-down, selecting a variable that optimally separates the set of objects at each step.

Instead of a single tree, some techniques, often called ensemble methods, construct more than one DT. They are called boosted trees and bagged DT (Breiman 1996; Friedman 1999). Boosted trees mainly aim to decrease bias, whereas the objective of bagging trees is to decrease variance (Rokach and Maimon 2005).

### Random forest

This method, which is a bagging ensemble technique, brings the predictions of multiple DTs (outcomes) together and makes predictions based on the average values of the predictions of these trees. The first step is to choose a subset of the dataset, and then the separate DT with a randomly selected subset of features is built in parallel. Unlike DT, root and separated nodes are randomly selected here. As might be expected, as the number of trees increases, the accuracy is improved. One essential hyperparameter in this algorithm is the number of estimators, representing the number of trees in the forest. Grid search cross-validation is often applied to find the best number of estimator values. One important advantage of RF algorithms is that they cause fewer overfitting problems.

### Extreme gradient boosting algorithm

One other ensemble supervised machine learning method is gradient boosting developed by Chen and Guestrin (2016). It is a quick, efficient algorithm and is gaining very high popularity in the machine learning area. Unlike RF algorithms, in XGBoost, the diverse DTs are run sequentially, not in parallel. In this algorithm, trees are added individually to the group, and prediction mistakes of the past models are corrected. Here the gradient descent algorithm is used to minimize the loss gradient. Several hyperparameters of XGBoost method are as follows:

- *colsample_bytree*: the ratio of columns while constructing a tree;
- *gamma*: the overfitting control parameter;
- *max depth*: used to control the tree depth;
- *reglambda*: the L2 regulator for leaf weights;
- *eta*: the learning rate used while minimizing the cost function.

### Multi-layer neural networks

**Fig. 1:**
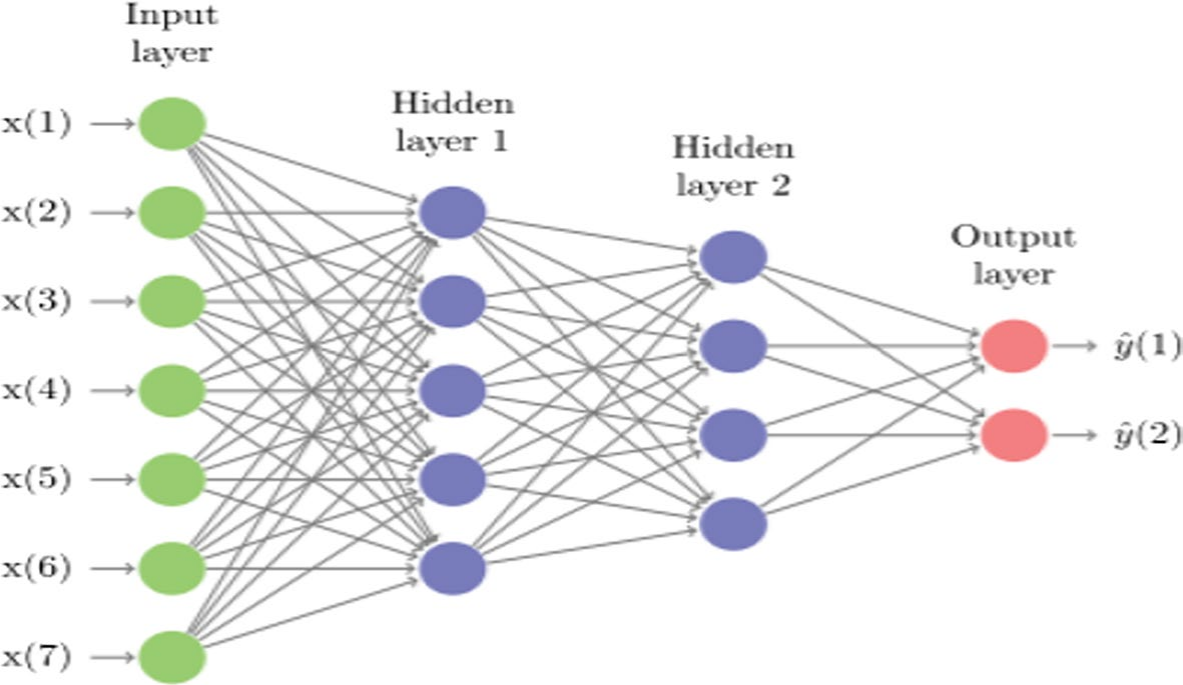
The representation of a multi-layer neural network (Dixon et al. 2017). The input layer consists of explanatory variables called features, and the information is forwarded from this layer to the hidden layers. On the arcs of hidden layers, parameters called weights and biases exist. The goal of the network is to find the optimal parameter settings that minimize the error between the estimated and the actual target value

This method is developed by Rumelhart et al. (1986) and forms the basis of deep learning studies. These networks consist of an input layer, at least one hidden layer, and an output layer, and each layer is made up of a set of units (neurons). The layers are fully connected (dense), which means that all input units from one layer are connected to every activation unit of the succeeding layer (Fig. 1). The network computes the prediction through forward propagation with several activation functions and minimizes the error through backward propagation by modifying the network weights and biases to set up the optimal parameters for the prediction.

## Empirical findings

**Fig. 2:**
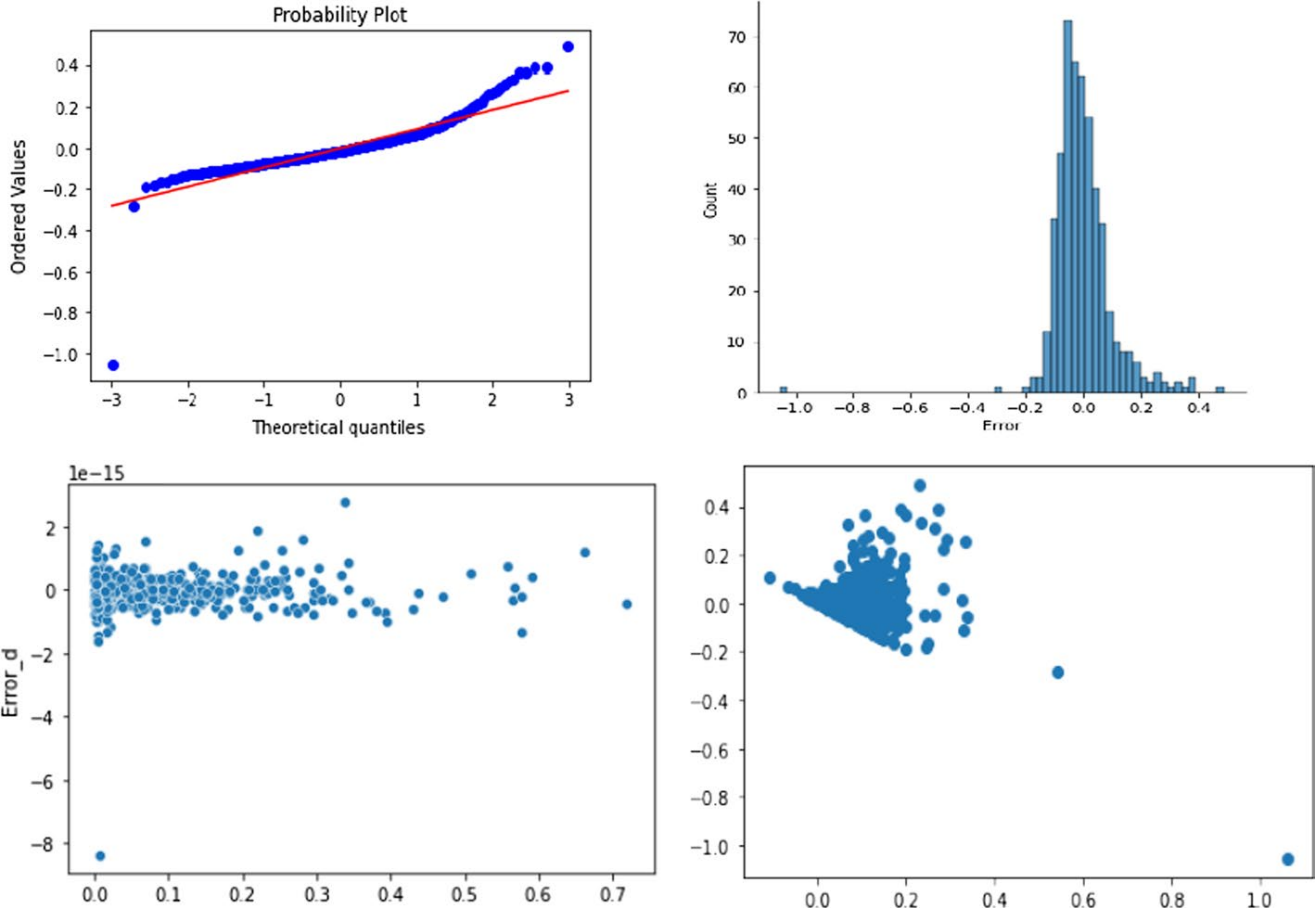
Plots showing the regression assumptions for the model. Errors are approximately normally distributed (2.1). The mean of errors is approximately zero (2.2). This shows that homoscedasticity (errors almost have equal variances) holds (2.3). Outliers are negligible (2.4)

In this study, we try predicting the cash holdings of firms using several supervised machine learning techniques. To make a good prediction for CASH using Python software, the authors evaluated all supervised learning regression methods discussed in the methodology based on the error metric RMSE and test data R^2^. RMSE is a function of the differences between the observed and predicted values. Therefore, lower RMSE values of regression models are expected. Meanwhile, R^2^ shows how well the regression model fits the observed values of the dependent variable. Therefore, higher R^2^ values are desired. To prevent the overfitting problem causing poor predictions with the unseen data, we split 80% of the dataset as training data and take the remaining 20% as test data. First, MLR is used to predict CASH under various predictor variables. To apply multiple regression, we checked the assumptions in Sect. 3.2.1. Figure 2 shows that errors are normally distributed, and the relationship is linear.

The pairwise correlation matrix and VIF results are presented in Table 2. VIF shows the multicollinearity problem among the independent variables. If the VIF is greater than 5 or 10, multicollinearity is deemed high in the respective regression models (Guizani 2017). The mean VIF is 1.50, indicating no multicollinearity problem among the variables.

**Table 2:** Variance inflation factor

Variables	VIF
STD	2.34
CF	2.30
IE	2.28
NWC	2.26
ROA	1.63
PRETAXMARGIN	1.60
ROIC	1.58
NETMARGIN	1.58
ROE	1.51
PPE	1.38
SIZE	1.33
CR	1.26
AR	1.26
DIV	1.23
AGE	1.17
EPS	1.13
WUI_TURKEY	1.09
CAPEX	1.08
SG	1.03
AP	1.02

Performance metrics after applying MLR are displayed in Table 3. RMSE is high and R^2^ is low; the performance metrics with those 15 features in the model are shown in Table 3. Additionally, the most correlated 15 features are screened out (Table 4). The results are still found to be unsuccessful; therefore, we can conclude that MLR is not good at predicting CASH values***.***

**Table 3:** Performance metrics with MLR

MLR	Model with 19 features	Model with 15 features
RMSE	0.1109	0.1036
R^2^	0.0406	0.1626

**Table 4:** Correlation between features and CASH

Variable	Correlation coefficient
CASH	1.0000
CR	0.3556
TANG	0.2851
CF	0.2363
DIV	0.2356
EPS	0.2223
STD	0.1717
IE	0.1679
SIZE	0.1660
ROIC	0.1233
AR	0.1112
PRETAXMARGIN	0.0861
ROA	0.0783
NETMARGIN	0.0578
AP	0.0559

Fitting a regression model with more than 15 explanatory variables is difficult; thus, the MLR model is modified and rerun with five or six independent variables that are lowly correlated with each other (Table 5). Based on these models, the R^2^ and RMSE values do not improve; therefore, MLR is not an appropriate algorithm for this prediction.

**Table 5:** Performance metrics for MLR with various predictors

Model no	Model predictors	R^2^	RMSE
1	CR, TANG, CF, DIV, EPS, STD	0.1611	0.1037
2	NWC, CR, SIZE, CF, Age	0.161	0.1036
3	Age, WUI, CAPEX, DIV, IE	0.0755	0.1109
4	CF, SIZE, NWC, SSG, STD	0.1022	0.1093

**Fig. 3:**
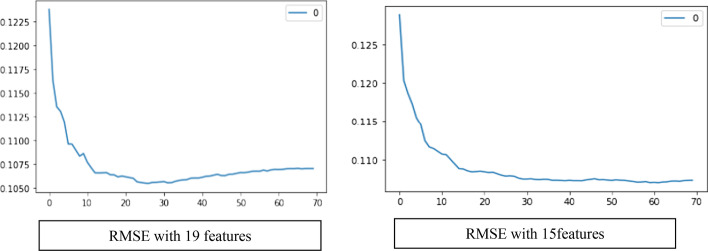
RMSE with different k settings. With all 19 features, the best hyperparameter k is 27, whereas with reduced 15 features, the best k is 57

Another algorithm, KNN, is also applied to predict CASH value with several predictor variables. To find the best k value that minimizes model error, we applied the grid search cross-validation (Fig. 3) and chose 27 as the optimal k. The optimal k value with the chosen 15-features model (Table 4) is found to be 57.

As shown in Table 6, RMSE results for both models are still high, and R^2^ value is low. Therefore, KNN is unsuccessful in predicting CASH values, although it gives improved results compared with the MLR model.

**Table 6:** Performance metrics with KNN

KNN	Model with 19 features	Model with 15 features
k	27	57
RMSE	0.1064	0.1071
R^2^	0.1071	0.1228

The SVR algorithm is the third supervised machine learning algorithm for CASH prediction. With grid search cross-validation, the hyperparameter C value is 2. Table 7 shows that the RMSE value is still not very low, whereas the R^2^ value is low. However, SVR provides much better performance metrics compared with MLR and KNN algorithms for predicting CASH.

**Table 7:** Performance metrics with SVR

SVR	Model with 19 features	Model with 15 features
RMSE	0.0796	0.0887
R^2^	0.5152	0.3984

Thereafter, the DT algorithm is applied for the CASH prediction. For this algorithm, the optimal maximum tree depth (max depth) parameter is 5. The number of features used in this algorithm decreases based on the descending correlation scores, and those new models are also run. Based on Table 8, RMSE values are larger than SVR algorithm outputs, and R^2^ is lower. Therefore, the DT algorithm is also not good at predicting the response variable CASH.

**Table 8:** Performance metrics with decision trees

Decision trees	Model with 19 features	Model with 15 features	Model with 10 features	Model with 5 features
Max depth	5	5	5	5
RMSE	0.0906	0.0915	0.0903	0.0899
R^2^	0.3723	0.3756	0.3812	0.3822

RF is the next algorithm used for CASH prediction. Grid search cross-validation provides the optimal number of estimators (n_estimators) differing as to the number of features. As shown in Table 9, RMSE values are lower compared with the previous algorithms, and R^2^ values are higher. Moreover, as the number of features decreases, these two metrics improve.

**Table 9:** Performance metrics with RF

Random forest	Model with 19 features	Model with 15 features	Model with 10 features	Model with 5 features
n_estimators	600	1000	1000	1000
RMSE	0.0722	0.0718	0.0713	0.0710
R^2^	0.6016	0.6054	0.6111	0.6147

Penultimately, the XGBoost algorithm for CASH prediction is applied. This algorithm has various hyperparameters, and grid search cross-validation finds the optimal settings for the selected hyperparameter set. Some important hyperparameters are “colsample by tree,” which is the fraction of columns when constructing a tree, “n estimators,” which is the number of trees in the model, “gamma,” which is the regularization parameter for a minimum loss reduction, “max depth,” which is the maximum tree length from node to leaves, “reg lambda,” which is the L2 regularization term, and “eta,” which is the learning rate. Optimal hyperparameters are displayed in bold in Table 10.

**Table 10:** XGBoost best parameter setting

Colsample by tree	0.5	0.6	0.7	0.8	0.9	**1**
n estimators	500	600	**700**			
Gamma	0	**1**				
Max depth	3	**4**	5			
Reg lambda	1	**1.5**				
Eta	0.01	0.05	**0.1**			

The hyperparameter values displayed in bold are the best settings that provide the maximum R^2^. The optimal hyperparameter setting is obtained by assigning the following values: colsample by tree = 1, n estimators = 700, gamma = 1, max tree depth = 4, reg lambda i = 1.5, and eta = 0.1

Table 11 shows that the XGBoost algorithm yields the lowest RMSE and highest R^2^ among all applied machine learning methods in this study. The model captures 73% of the observed variability in the CASH values. When the number of features used in the model is decreased, the model outcome values deteriorate significantly. Therefore, the model with all features included is chosen as the best model to predict the response variable CASH.

**Table 11:** Performance metrics with XGBoost

XGBoost	Model with 19 features	Model with 15 features	Model with 10 features	Model with 5 features
RMSE	0.0599	0.1000	0.0991	0.0990
R^2^	0.7258	0.2340	0.2488	0.2495

**Fig. 4:**
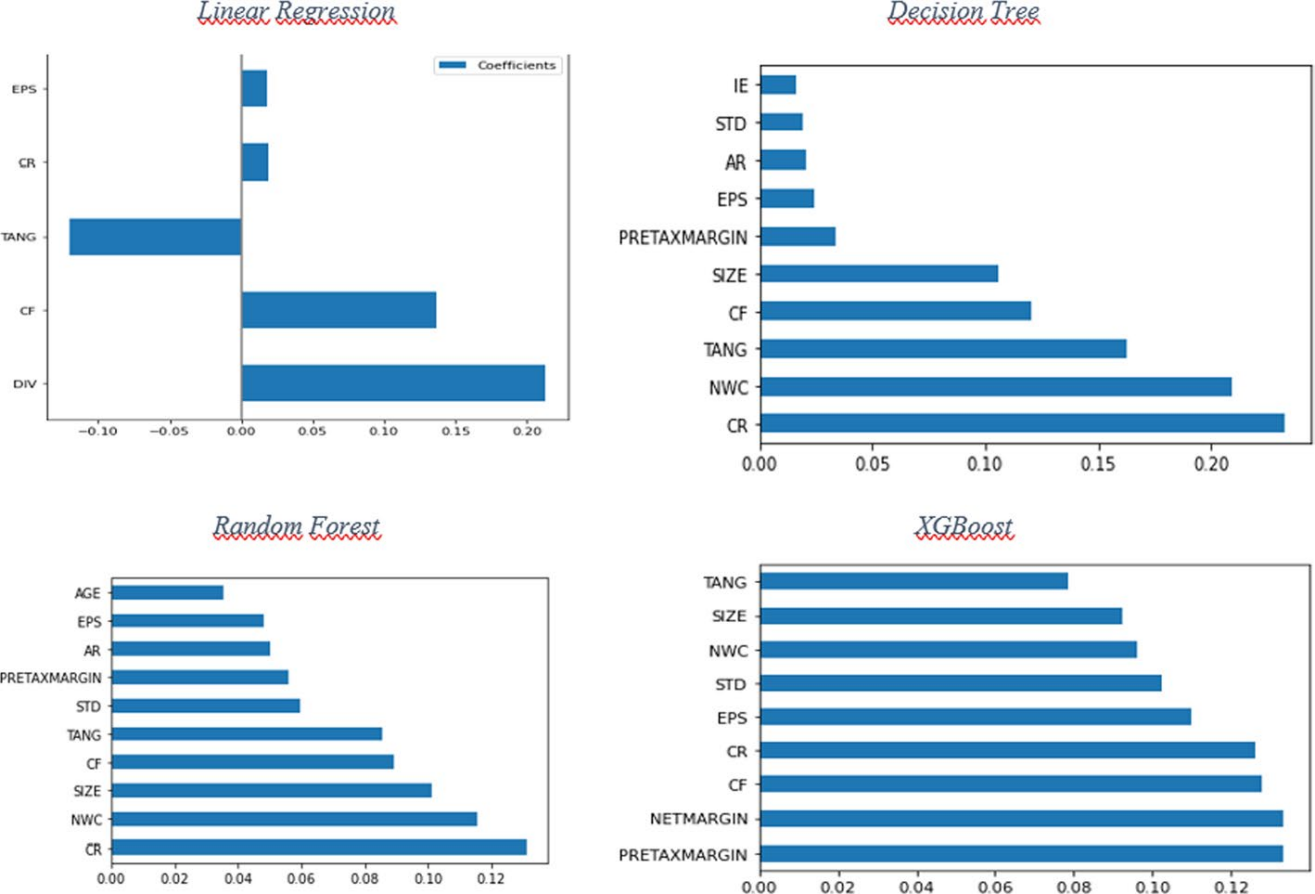
Feature importance bar charts for several machine learning algorithms

XGBoost also provides the feature importance plot that shows the most dominant features used in the model (Fig. 4). The most fundamental features providing a high-performance model include pre-tax margin, net margin, cash flow, and current ratio. Lastly, the deep learning algorithm multi-layer neural network (MLNN) is used for CASH prediction. This algorithm’s best hyperparameter setting includes three to five dense hidden layers with 64 nodes in Table 12. The model outputs with high RMSE, and low R^2^ indicates that this model is unsuccessful in predicting CASH values.

**Table 12:** Performance metrics with MLNN

MLNN	Model with 19 features	Model with 15 features	Model with 10 features	Model with 5 features
Hidden layers	3	5	5	5
RMSE	0.1016	0.1086	0.0991	0.1136
R^2^	0.2105	0.0974	0.060	0.0121

In summary, first, less complex machine learning methods are applied to the dataset, starting with MLR. The assumptions are checked, and MLR results yield poor performance metrics (i.e., high RMSE and low R^2^ values). The KNN and SVR models are also applied, and the results show that neither model improves the performance metrics. Then, tree-based machine learning techniques, such as DT, RF, and XGBoost algorithms, are ran with the dataset, improving the prediction capability considerably. With the DT, RF, and XGBoost, the R^2^ values increase to 0.38, 0.61, and 0.73, respectively, by involving all 20 features. To check whether a smaller number of features improve the results, this study selected 15, 10, and 5 features, respectively, with high correlation coefficients and modified the models. However, the DT and RF values slightly changed, whereas the XGBoost shows a significant reduction in R^2^ values. Therefore, the XGBoost model with all 20 features is the best regressor for CASH prediction. The most dominant features are pretax margin, net margin, cash flow and current ratio. Lastly, MLNN is also applied with several hyperparameter settings, and yet the outcomes have not yielded good performance compared with the tree-based algorithms. Table 13 compares the applied supervised machine learning algorithms for CASH prediction. The best results are obtained using the XGBoost algorithm (0.06 RMSE and 0.73 R^2^ values). Compared with KNN which is the worst result-giving algorithm, XGBoost provides 42% lower RMSE value and 400% higher R^2^ value.

**Table 13:** Performance metrics comparison for the algorithms

	MLR	KNN	SVR	Decision trees	Random forest	XGBoost
RMSE	0.1036	0.1064	0.0796	0.0899	0.071	0.0599
% Improvement in RMSE	0	− 2.7027	23.1660	13.2239	31.4672	42.1815
R^2^	0.1626	0.1228	0.5152	0.3822	0.6147	0.7258
% Improvement in R^2^	32.4104	0	319.5440	211.2378	400.5700	491.0423

The algorithms applied in the study are evaluated based on the RMSE and R^2^ values. For the RMSE, the minimum value is obtained from the XGBoost algorithm. This RMSE is 42.18% lower than that of MLR algorithm, which is the highest. For R^2^, the maximum value is obtained by XGBoost algorithm again. This R^2^ is 42.18% lower than that of KNN algorithm, which is the lowest R^2^ value among all

Some machine learning algorithms, especially tree-based ones, provide the most dominant (important) features by using bar charts (Fig. 4). The three most influential variables for linear regression are dividend, cash flow, and tangibility, respectively. For DT algorithm, current ratio is the most important variable, followed by NWC and TANG. Similarly to DT, RF shows that current ratio is the most important feature, followed by NWC and TANG. Lastly, for the XGBoost algorithm, which gives the highest R^2^ value, Pretax Margin and Net Margin are the two most important features. The common features important for each of these four algorithms are current ratio, TANG, and NWC.

## Conclusions and discussions

The decision of firms to hold cash is a popular subject in modern corporate finance. The fact is that firms maintain a considerable amount of cash for various purposes, such as financing growth, paying taxes, or retiring matured debts. In this study, we try predicting the firm’s cash holdings using several supervised machine learning regression techniques. The study considers 211 BIST listed firms from 2006 to 2019. The dataset has 19 firm-level financial variables and a country-specific WUI for Turkey. MLR, KNN, SVR, DT, RF, XGBoost, and MLNN are used for prediction. The results show that as we proceed with more advanced algorithms, considerable improvements are observed with a maximum of 42% improvement in RMSE values (vs. KNN, the worst-performing algorithm) and more than 400% improvement in R^2^ values). The XGBoost algorithm yielded the best results.

The findings imply that the most dominant features are cash flow, current ratio, pretax margin, and net margin. Cash flow is an important item for firms because the tendency to hold cash depends on whether cash flow is high or low. Based on the financial hierarchy theory, internal finance is strongly preferred by firms that believe its cost advantage over debt and equity (Myers 1984). Related to this theory, Ferreira and Vilela (2004), García-Teruel and Martínez-Solano (2008), Ozkan and Ozkan (2004), and Uyar and Kuzey (2014) identified a positive relationship between cash holdings and cash flow. However, Chen (2008), Kim et al. (1998), and Kim et al. (2011) found a negative relationship between cash holdings and cash flow, claiming that cash flow provides an additional source of liquidity, and it can be used as a cash substitute. Firms with high cash flow may prefer to hold less cash, whereas firms with low cash flow prefer to hold more cash to meet investment opportunities. Additionally, firms are motivated to keep investment activities and reduce their cash holdings during stable periods (Chiu et al. 2016). However, as uncertainty increases in local and global markets, firms want to retain more cash on hand to mitigate investment risks (Gulen and Ion 2016). As Opler et al. (1999) stated, cash holdings as a precautionary measure are an efficient strategy for firms to manage the turbulence in the internal and external environments.

The current ratio measures the firm’s capability to meet short-term obligations due within one year. This gives a signal about the financial health of the company. Meanwhile, net margin provides information on how much profit company generates for each dollar of revenue it generates. Angelovska and Valentinčič (2019) find that an increase of one standard deviation leads to an average 41.5% increase in cash.

Based on our findings, this study has significant implications for corporate managers and researchers. Managers can use this information to determine the firms’ cash holdings for making corporate policies. Meanwhile, researchers can use the information to create better regression models and find the cash holdings behavior of companies.

This study also has some limitations. We focus mainly on Turkish firms and their characteristics. The period of the study is between 2006 and 2019. In further studies, the period can be expanded, and macroeconomic variables, such as gross domestic product growth, interest rates, and oil prices, can be added to the studies.

Moreover, the factor of sector classification is not added, and firms can be analyzed on the basis of their sectors in future studies. Besides the time span, the number of countries can be expanded. Future studies can consider a cross-country analysis. For example, researchers can predict cash holdings for developed and emerging markets to determine whether any differences exist in cash holdings levels between markets. They can also compare firms in different continents to find any regional differences in the impacts on cash holdings levels. Finally, because of COVID-19 effect on financial variables in 2020, this study excludes the 2020 variables of Turkish firms. Researchers can also include the COVID-19 effect on cash holdings levels for their future studies.

## Data Availability

The data that support the findings of this study are available from Thomson Reuters DataStream. Restrictions may apply to the availability of these data, which were used under license.
